# Real-Time Observation of Nanoscale Kink Band Mediated Plasticity in Ion-Irradiated Graphite: An In Situ TEM Study

**DOI:** 10.3390/ma17040895

**Published:** 2024-02-15

**Authors:** Melonie P. Thomas, Ryan Schoell, Nahid Sultan Al-Mamun, Winson Kuo, John Watt, William Windes, Khalid Hattar, Aman Haque

**Affiliations:** 1 Department of Mechanical Engineering, The Pennsylvania State University, University Park, PA 16802, USA; mkt5693@psu.edu (M.P.T.); nma5621@psu.edu (N.S.A.-M.); 2 Center for Integrated Nanotechnologies, Sandia National Laboratories, P.O. Box 5800, Albuquerque, NM 87185, USA; rmschoe@sandia.gov; 3 Center for Integrated Nanotechnologies, Los Alamos National Laboratory, Los Alamos, NM 87545, USA; kuo@lanl.gov (W.K.); watt@lanl.gov (J.W.); 4 Idaho National Laboratory, Idaho Falls, ID 83415, USA; william.windes@inl.gov; 5 Department of Nuclear Engineering, University of Tennessee, Knoxville, TN 37996, USA; khattar@utk.edu

**Keywords:** layered solids, graphite, heavy ion irradiation, shear banding, ripplocations, kink bands, in situ transmission electron microscopy, nanoindentation

## Abstract

Graphite IG-110 is a synthetic polycrystalline material used as a neutron moderator in reactors. Graphite is inherently brittle and is known to exhibit a further increase in brittleness due to radiation damage at room temperature. To understand the irradiation effects on pre-existing defects and their overall influence on external load, micropillar compression tests were performed using in situ nanoindentation in the Transmission Electron Microscopy (TEM) for both pristine and ion-irradiated samples. While pristine specimens showed brittle and subsequent catastrophic failure, the 2.8 MeV Au^2+^ ion (fluence of 4.378 × 10^14^ cm^−2^) irradiated specimens sustained extensive plasticity at room temperature without failure. In situ TEM characterization showed nucleation of nanoscale kink band structures at numerous sites, where the localized plasticity appeared to close the defects and cracks while allowing large average strain. We propose that compressive mechanical stress due to dimensional change during ion irradiation transforms buckled basal layers in graphite into kink bands. The externally applied load during the micropillar tests proliferates the nucleation and motion of kink bands to accommodate the large plastic strain. The inherent non-uniformity of graphite microstructure promotes such strain localization, making kink bands the predominant mechanism behind unprecedented toughness in an otherwise brittle material.

## 1. Introduction

Layered crystalline solids are commonly found in nature, although some are synthetic. These solids have a wide range of applications in fields such as electronics, optics, energy storage, and environmental remediation. Their size can vary over ten orders of magnitude. Graphite is a layered crystalline solid made up of parallel two-dimensional graphene sheets. In these sheets, sp2 hybridized carbon atoms are arranged in hexagonal rings. The graphene sheets in graphite are separated by a distance of 0.335 nm and are held together by weak van der Waals forces, which gives the material its soft and sliding nature [1,2,3]. Synthetic graphite has been used in nuclear reactors since the first reactor, Chicago Pile-1 (CP-1), and is expected to be important in next-generation high-temperature reactors and molten salt-cooled reactors [4]. It is a complex polycrystalline multicomponent material made of petroleum coke as filler and coal-tar pitch as binder and graphitized at very high temperatures. Graphitization improves neutron moderation and oxidation performance [5] but also creates defects spanning atomic to sub-mm length scales. Grain size is usually inferred from the filler particle size, which contains nanoscale crystallites containing stacking faults in one layer for approximately every six layers [6]. Cracks are frequent features in polycrystalline graphite, with sizes ranging from a few nanometers to tens of micrometers [7]. Particularly notable are nanoscale Mrozowski cracks that form between the basal planes during post-graphitization cooling [8]. Poor graphitization can result in nanoscale rosette-like structures known as quinoline-insoluble particles [9]. Volumetric shrinkage during cooling also gives rise to much larger intergranular cracks along the interfaces and is not necessarily related to the basal planes. Finally, gas-escape pores of a much larger scale develop during the removal of volatiles during the manufacturing process. The voids and cracks can occupy up to 20% of the total volume [10].

The objective of this study is to examine the mechanical behavior of graphite with a focus on the role of nanoscale defects. Thermo-mechanical properties are strongly influenced by the size and distribution of these microstructural features, making structure–property correlation quite complex [11,12]. For example, the microscale high-aspect ratio intergranular cracks promote internal stress concentration, yet nanoscale Mrozowski cracks with similar aspect ratios help densification during irradiation [13]. The conundrum is that defects in polycrystalline graphite are helpful in accommodating deformation during external loading and radiation but also contribute to oxidation and damage at higher doses [14]. The larger pores influence mechanical properties, while smaller pores control irradiation-induced dimensional changes. Advanced techniques, such as X-ray computed tomography, enabled probing microstructure–property relationships [15,16,17] at the microscale. Transmission Electron Microscopy (TEM) is uniquely suitable to visualize submicron scale defects [10,18,19,20], but it can be challenging to conduct in situ property measurements in the TEM [21]. TEM has enabled significant progress in atomic and nanoscale microstructural defects unique to layered materials. For example, ruck and tuck features are unique defects that can explain radiation expansion of the c-axis as an alternative to conventional additional basal plane formation by dislocation loops [22,23]. Ripplocation boundaries, derived from atomic scale ripples, are also reported to play a dominant role in deformation mechanisms in layered materials [24,25].

In this study, we focus on the submicron mechanical behavior of graphite, IG-110, in both pristine and irradiated conditions. At the macroscale, polycrystalline graphite is brittle with slight non-linear behavior, which tends to be more linear and brittle upon irradiation [6]. Depending upon the dose, neutrons create defects ranging from vacancies and interstitial pairs to larger and more complex three-dimensional structures [26] or, in extreme cases, amorphization [27]. Fresh nuclear-grade graphite contains large basal dislocation density with higher mobility, and, upon irradiation, the materials’ stiffness is enhanced by dislocation pinning [28]. A ‘turnaround’ dose exists below in which there is a negative volumetric change due to c-axis expansion and a-axis shrinkage. Such densification manifests in the closing of the finer pores, increasing both stiffness and strength. These values can be up to twice the value of the pristine material [28,29,30]. Above the turnaround dose, the mechanism to accommodate a-axis shrinkage desists while new radiation-induced defects lower the toughness of the material [13,31,32]. Temperature is known to influence these mechanisms by in situ annealing [16,26] or creating specific defect types as well as oxidation [33,34]. It is also important to note that ion irradiation is frequently used as a surrogate of neutron irradiation because of its ability to produce high levels of displacement damage compared to neutron irradiation [35,36]. Ion beam irradiation can cover a broad range of the neutron recoil spectrum at significantly higher dose rates without any radioactivity in the specimens under most conditions [36].

It is important to note that most of the reported studies of mechanical testing on polycrystalline graphite were performed at the macroscopic length scale. The literature adequately addresses the needs of defect and microstructure characterization, but does not connect them to the mechanical behavior, particularly at the submicron length scale. One study of particular significance to the smaller length scales is by Liu et al. [12], who performed nanoindentation (indentation depth varied up to 1 micron) and cantilever beam bending experiments at the micro (2 × 2 µm^2^) and meso (20 × 20 µm^2^) scales. These findings show that stiffness and strength increase with decreasing length scale, but so does the scatter in the data. The authors reported higher failure strain at these length scales compared to the macro-scale and deduced that the millimeter-sized pores contribute to the ‘brittleness’ at the macro-scale. Nanoindentation allows highly localized force to be applied, and follow-up microscopy shows that graphite can deform via buckling with kink formation at higher temperatures [37]. In situ mechanical testing with nanometer resolution could be more beneficial in understanding the fundamental deformation mechanisms. However, the only published instance of such an experiment was limited to strain mapping only [21]. Therefore, a significant opportunity exists to perform in situ TEM mechanical tests on nanoscale graphite specimens. A fundamental understanding of mechanical behavior at this scale could shed light on the diverse mechanisms reported in the literature, germinate new concepts on radiation-induced plasticity, and expand our understanding of the multi-scale mechanisms probably active in graphite during thermal, mechanical, and irradiation conditions.

## 2. Materials and Methods

**Figure 1:**
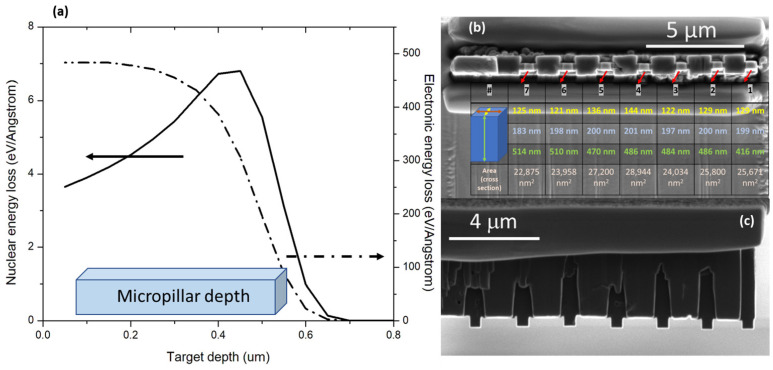
(**a**) SRIM simulated electronic (dashed line) and nuclear (solid line) energy loss profiles as a function of penetration depth for 2.8 MeV Au ions. (**b**) Scanning Electron Microscopy (SEM) micrographs showing end view of the irradiated set of micropillar compression testing specimens fabricated by FIB-SEM. The tables include nominal values of varying dimensions of each pillar and measured by SEM and TEM micrographs. The yellow measurements indicate the side of the pillar parallel to the electron beam of the TEM. (**c**) Top view of the specimens.

Fine-grained graphite IG-110 (Toyo Tanso Co., Kagawa, Japan) was used in this study in both pristine and ion-irradiated forms. This material is well studied in the literature with developed mathematical models [38] and is used in high-temperature gas-cooled reactors. The bulk material was irradiated at the Ion Beam Laboratory at the Sandia National Laboratories using the 6 MV Tandem accelerator. We used a 2.8 MeV Au^2+^ beam with a fluence of 4.378 × 10^14^ cm^−2^ (~peak 8.8 dpa) at 25 °C. The nuclear and electronic energy loss distribution resulting from the 2.8 MeV Au, as predicted by the Stopping and Range of Ions in Matter (SRIM) code in “Ion Distribution and Quick Calculation of Damage” mode and displacement energy of carbon atom and the surface binding energy for sputtering was set to 28 eV and 7.4 eV, respectively, as seen in Figure 1a. Micro-pillars were fabricated from both pristine and irradiated samples using a Scios 2 Dual Beam Scanning Electron Microscope–Focused Ion Beam (SEM-FIB) (Thermo Fisher Scientific-Albuquerque, NM, USA) to obtain the cuboid shape of each pillar and to achieve electron transparency of specimens. The pillars were shaped to be approximately 450 nm tall, 200 nm wide, and 130 nm in thickness. The cuboid pillar geometry was chosen to obtain a good balance between the electron transparency of the specimen to detect any defects and to minimize the contribution of buckling or bending upon applied force. Figure 1b,c show a scanning electron micrograph of the end and top views of micro-pillar specimens, respectively. The detailed FIB procedure for micropillar fabrication is given in Figure S1 (refer to Supplementary Information). A total of eleven specimens (four pristine and seven irradiated) were tested.

**Figure 2:**
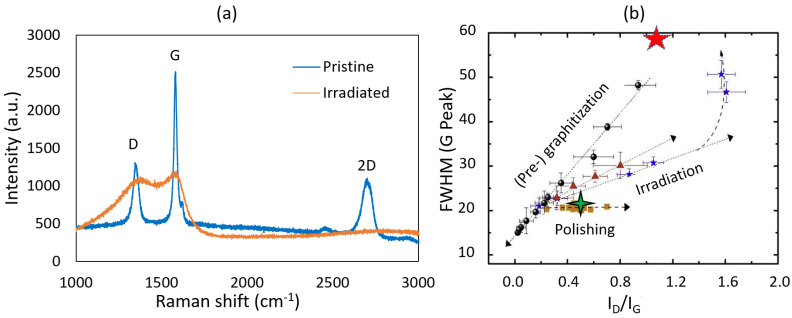
(**a**) Raman spectroscopy performed on the pristine and ion-irradiated IG-110 specimens (**b**) comparison of the FWHM and I_D_/I_G_ parameters compiled in the literature for various polycrystalline graphite samples (adapted from [40]).

**Figure 3:**
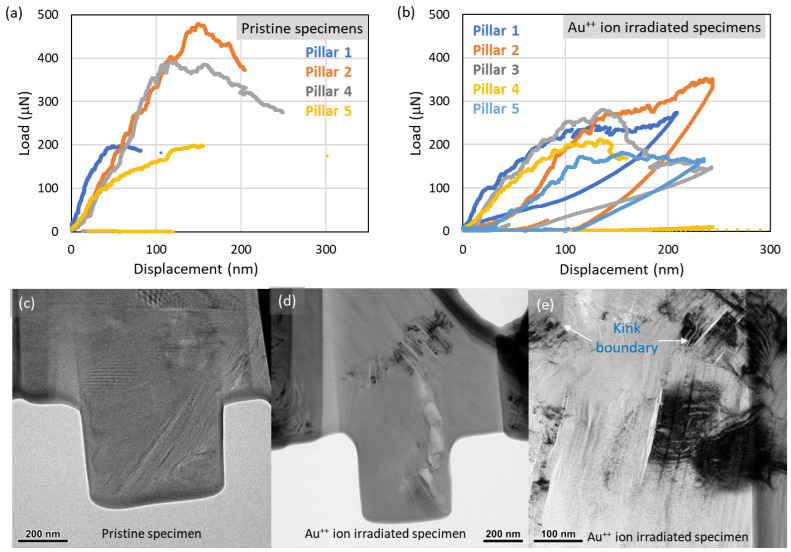
Micropillar compression testing results (load-displacement data) for (**a**) pristine and (**b**) ion-irradiated IG-110 graphite specimens. TEM bright-field micrographs of micropillars showing the microstructure of (**c**) pristine and (Au-ion irradiated specimen prior to any mechanical loading. (**e**) High-resolution bright-field TEM micrograph of (**d**) with kink bands and kink boundaries labeled with white arrows.

To assess the extent of radiation damage, Raman spectroscopy characterization was performed on both pristine and irradiated graphite specimens using a Horiba LabRam HR Evolution confocal Raman microscope (Figure 2a). An average Duoscan spot diameter of 20 µm was utilized to reduce the effective power density of the laser and to increase the measured Raman spectral response. Raman spectral data were obtained using a 785 nm wavelength laser with laser power maintained at approximately 1.7 mW, a 300 gr/mm grating, and a confocal hole size of 200 µm. Acquisitions were recorded with 60 s integration and 3 accumulations. Lorentzian peak fitting of the Raman spectral data was performed using the software Origin, version 2020. Figure 3a compares the intensity ratio of the defect-induced (D) and the symmetry-allowed (G) bands. As shown in Figure 2b, the intensity ratio (I_D_/I_G_) increases with radiation damage due to increased defect density. The Full Width at Half Maximum (FWHM) of Raman spectral peaks is an indicator of degradation in crystallinity due to irradiation [39]. Both these parameters are needed because the D intensity can independently vary with different types of defects. Figure 2b shows the data for the pristine (4-pointed star) and irradiated (5-pointed star) specimens in this study. The pristine specimens showed a FWHM of 21.42 and an I_D_/I_G_ ratio of 0.52. These values changed to 120.92 and 0.92, respectively, after irradiation. The 5-pointed star in Figure 2b is therefore located in the correct x-coordinate but could not be fitted in the y-coordinate. This suggests disruption of crystalline order in very large order [40].

Compression testing of the micropillars was performed in an image-corrected and mono-chromated FEI Titan G2 Environmental Transmission Electron microscope (ETEM) at 300 kV. Load and displacement were applied and measured using the Bruker/Hysitron PI-95 TEM Picoindenter system. The PI-95 1 µm conical flat punch was used for all compression tests and the tests were run in displacement-controlled mode by setting the displacement depth to half of the initial height of each pillar (the displacement rate was set to ~0.5 nm/sec). Load-displacement measurements and TEM video of microstructural changes were recorded for each pillar compression test.

## 3. Results and Discussion

Figure 3 shows the loading and unloading results for the pristine and ion-irradiated specimens. All pristine specimens failed catastrophically, as expected for a brittle material. On the other hand, none of the ion-irradiated specimens failed. Rather, they showed remarkable plasticity and bulged before unloading. The specimen-to-specimen variation in the load-displacement data in Figure 3 arises from the extreme difficulty of perfectly aligning the nanoscale flat punch on the cuboid surface and is effectively unavoidable for pillar compression experiments. Therefore, our interpretation of this data is semi-qualitative, as we focus more on the in situ deformation mechanisms. In addition to such specimen alignment error, appreciable variation is also expected [12] due to length-scale effects on defects and microstructure in graphite. This variation was enhanced in the irradiated specimens, which suggests that ion irradiation critically influences the pre-existing defects. However, Figure 3b indicates that ion-irradiated specimens in general (1) did not show radiation-induced hardening as typically seen in other ion- or neutron-irradiated specimens [28,29,30], (2) showed a decrease in stiffness, which is also a deviation from the literature, and (3) exhibited unusually high levels of plastic deformation, with some specimens showing ideal plastic behavior till the flat punch was retracted by the displacement control program in the experimental setup. The slight variation in load vs. displacement curves in Figure 3a,b could be attributed to the imperfect alignment of the pillar and indenter, minor deviation in pillar thicknesses (as stated in Figure 1b), and microstructural composition and defects in each pillar. The two isolated data points in Figure 3a for pillars 1 and 5 are due to the mechanical vibrations of the Nanoindenter after pillar failure (Supplementary Information: video files of pristine pillars).

The in situ TEM experiments also shed light on the deformation mechanisms and explain the unusual plasticity in the irradiated specimens. Figure 3c,d shows a TEM micrograph of the pristine and irradiated specimen before mechanical loading, respectively. Kink bands were readily observed in the irradiated specimen (Figure 3e), their boundaries indicated by the arrows. Kink bands are highly localized deformation regions in highly anisotropic layered systems, which can accommodate the strain energy by redistributing it from energetically expensive in-plane bonds to cheaper out-of-plane bonds [41]. Recent studies show the increasing appearance of kink bands [2,24,25,37,42] in layered solids, which may accommodate unprecedented amounts of plastic deformation in graphite beyond what is possible with conventional point defect models [18,23].

**Figure 4:**
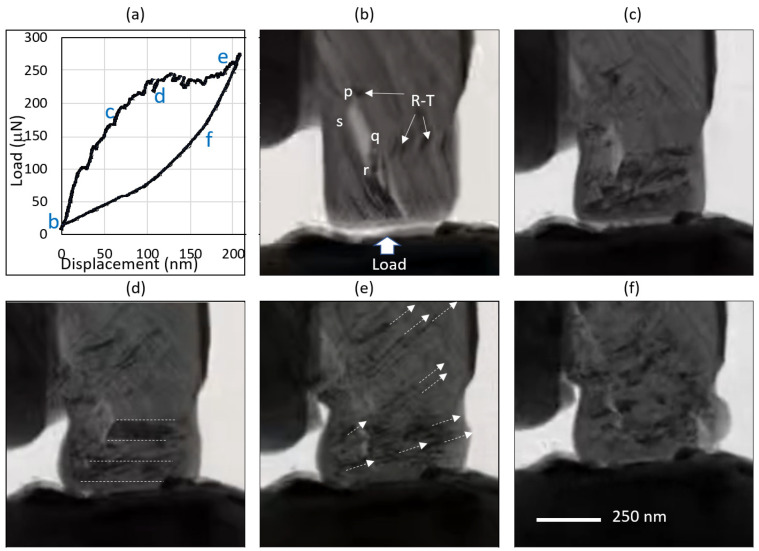
Snapshots from in situ TEM micropillar compression testing of an ion-irradiated IG-110 graphite specimen (pillar 1 of Figure 1b). (**a**) Load-displacement plot on which various load conditions are indicated: (**b**) load = 0 μN, (**c**) load = 190 μN, (**d**) load = 225 μN, (**e**) load = 275 μN, and (**f**) load = 150 μN upon flat punch retraction.

Figure 4 shows the TEM images of an irradiated specimen at different phases of the loading–unloading cycle. The magnification was intentionally kept low to minimize the electron beam damage, which can be quite severe. This was necessary as the low kV mode was unavailable for the TEM during the experiments. Nevertheless, the magnification was enough to observe submicron-scale mechanisms and to connect them to higher-magnification images before and after the experiments. Figure 4b shows the unloaded state of the ion-irradiated micropillar specimen, where conjugate delamination (marked as pqrs), as well as kink bands (marked with arrows), are observed. These two sub-micron defect features were commonly observed in our irradiated specimens. Also shown in Figure 4b is the vein-shaped ruck-tuck defect (marked as R-T) described in [23], only to be at the microscale. Further insights on the formation of these defects are given later in this section. Comparison of Figure 4b,c shows the remarkable feature of the kinks, which appear at the corners of the delamination defect. These are also the locations where stress concentrations are expected as the flat punch applies the compressive load on the specimen. Figure 4d shows the creation and propagation of new kink bands that resist deformation in the horizontal direction. These are indicated with dashed lines for ease of identification. Interestingly, these kink bands initially move more in a horizontal manner, possibly because of the predominance of the Hertzian contact stress. With the increased load on the flat punch, we see numerous nuclei of kink bands emanating randomly from the specimen (Figure 4d) and moving at about a 45° angle to the direction of compressive load. This is in agreement with the analysis described in [23], where a kink band ‘slip system’ seems to be active in the graphite specimen. Higher-magnification imaging of these black dots and bands indicates these to be multiples of kink bands piled against each other in a manner analogous to dislocation pile-ups in the grain boundaries of metallic materials. Kink band nucleation is not as random as it appears; rather, it is due to the spatial and size variation of the strength of the defects. The nucleation of the kink bands can also be dictated by the large residual stress from the harsh manufacturing process that remains frozen in highly localized states in graphite [16]. It is known that spatial variation in the strength of materials leads to deformation localization [43]. Figure 4e shows the specimen under the highest applied load of 275 µN. A significant increase was observed in these ‘slip lines’ and the disintegration of some of the wider (marked with dashed lines in Figure 4d) kink bands to narrower ones to store the high-strain energy. Finally, Figure 4f shows the irreversible nature of this deformation, where these kink bands remain mostly frozen upon load retraction. The pristine specimens did not show this mode of deformation at all; rather, all of them failed catastrophically.

**Figure 5:**
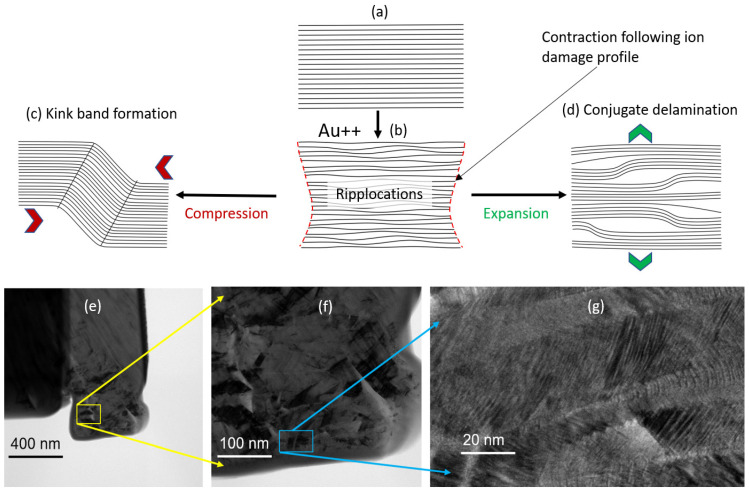
Schematic of kink band formation and conjugate delamination in graphite due to ion irradiation: (**a**) pristine graphite, (**b**) contraction in basal layers due to depth-dependent ion damage, resulting in ripplocations, (**c**) kink band formation under further compression, (**d**) conjugate delamination under further expansion. (**e**–**g**) TEM images from the plastically deformed irradiated specimen showing the kink bands at various magnifications.

While the density of the activated ‘slip planes’ varied from specimen to specimen, their presence is consistent and dynamic in all our irradiated specimens. A video from one of these specimens is available in the Supporting Information. While Figure 4 clearly shows the generation of the kink bands along the 45° slip plane, the video shows the unprecedented mobility of the kink bands along these planes. The unusually high strain rate observed in this study is difficult to explain through the conventional models using vacancies and interstitials. The findings of this study therefore support the ‘ripplocation’ model [44] of kink band formation, reproduced in Figure 5. The unusually high mobility of ripplocation boundaries (precursors to kink boundaries) makes them very sensitive to remote stresses, as well, which is evident in Figure 4. Ripplocation is the ripple-adapted version of dislocations, which are generated in layered solids under compression. The phenomenon is scale independent because it is derived from buckling. The pristine layers (Figure 5a) are compressed by the mechanical load arising from dimensional change due to displacing irradiation [45]. For ion irradiation, the dimensional change results in different degrees of basal plane contraction, according to the ion damage profile. This is shown in Figure 5b, where the contraction in the layers is schematically contracted to match an arbitrary ion damage profile. The localized buckling of the basal layers results in intermediate defects called ripplocations. Upon mechanical contraction, such as under an externally applied load or release of internal compressive residual stress, the ripplocations may transform into irreversible forms of kink bands. Conjugate delamination (marked as *pqrs* in Figure 4b) is also observed in the ion-irradiated specimens, which arise from expansion of the basal layers. Residual tensile stress or loading during specimen preparation process could lead to such events.

**Figure 6:**
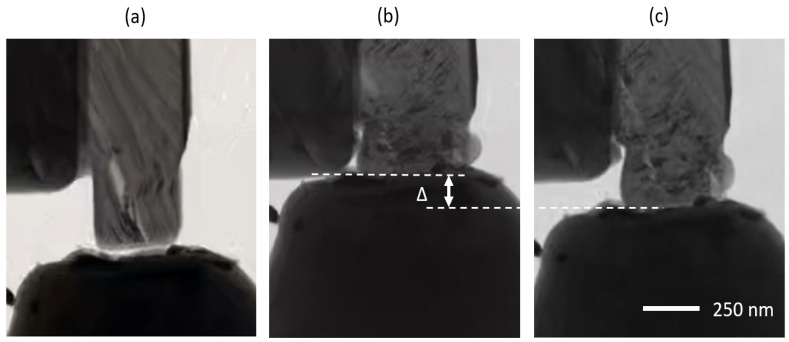
Irradiated micropillar specimen at different loading conditions, (**a**) P = 0 μN, (**b**) P_max_ = 275 μN, and (**c**) P = 0 μN after unloading.

Another unique aspect of the observed mechanical behavior of irradiated specimens is partial strain recovery upon unloading the flat punch. This is shown in Figure 6, where externally applied compressive load P = 0, P = P_max_ = 275 μN, and P = 0 after flat punch retraction scenario are presented. Interestingly, an appreciable amount of the total strain is recovered, even after the micropillar specimen remains bulged after unloading. This is indicated by the spring-back displacement of Δshown in Figure 6b. Comparison of Figure 6c,b clearly shows the recovered displacement even after a large amount of plastic deformation after complete unloading. Such recovery cannot be explained with the point defect model since basal dislocations are known to be permanent deformation. Interestingly, this observation can be explained by the ripplocation model, which is founded on the basis that ripplocations are elastic in nature until they are plastically transformed into kink bands. According to this model, it is possible that a certain fraction of the external compression is accommodated by ripplocations (and is, hence, recoverable) while another portion is plastic in nature (due to the formation of kink bands). The irreversible kink bands are still visible in Figure 6c, while the ripplocations (not seen at this magnification) are reversed and recovered.

An Important aspect of this study is the specimen size. The evidence of size effect on material behavior is abundant in the literature. In general, materials show higher strength at the micro- and nanoscales [46]. Anomalous behavior, such as plastic deformation of normally brittle materials is also reported [47]. Therefore, it is reasonable to assume that our experimental results show some size effects. However, examining both pristine and irradiated specimens at the same length scale allows us to draw a fair comparison to ascribe the observed plasticity and underlying deformation mechanisms to the effect of ion irradiation. An interesting extension of this work would be to study neutron-irradiated specimens to see the applicability at increasing in larger length-scale mechanical tests. Gray et al. have reported observed plasticity in the bulk nuclear graphite specimens in irradiation creep experiments. However, their deformation mode (creep at high temperature and longer time periods) is very different from the current study where we report kink band mediated plasticity without any influence of time or temperature [48].

**Figure 7:**
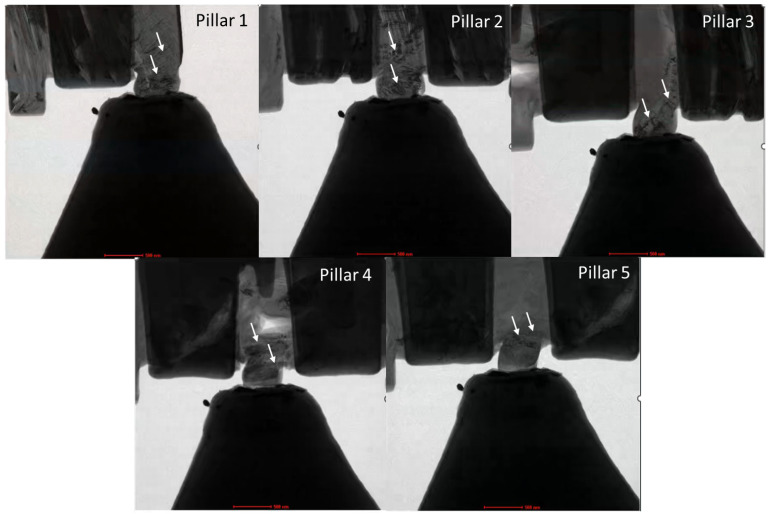
Kink-band mediated plasticity observed in all irradiated specimens from Figure 1b and Figure 3b. Kink bands are denoted with white color arrows. The scale bar is 500 nm.

The conceptual model or preliminary evidence of ruck-tuck, ripplocations, and kink bands is already available in the literature, but only for specimens that are not mechanically loaded. Therefore, the findings of this study translate these novel defect structures to a new level by visualizing deformation mechanisms in real time under externally applied mechanical loads. The unprecedented magnitude and rate of deformation as well as localization with the global effect of crack closure cannot be explained with the classical point defect models. The observed kink band mediated plasticity was observed in all the irradiated specimens as shown in Figure 7. Since nuclear graphite contains multi-scale defects, it is challenging to prepare two specimens with the same defect distribution, hence the mechanical properties could be different. Nevertheless, Figure 7 shows the consistency in the observed plasticity via kink banding in all the Au-ion irradiated specimens upon compressive load. Further information can be found in supplementary videos provided for each indentation experiment.

From an experimental perspective, future works could be planned to shed light on the influence of (1) radiation dose and ion species, (2) mechanical loading rate, (3) impact of sample size, and, more importantly, (4) specimen composition—such as filler or binder as the origin. Coupled simultaneous stressors like temperature or ion irradiation with mechanical loading can be used to study thermal creep or irradiation-induced creep, respectively. Both are important parameters for polycrystalline graphite and could also be studied in situ [49,50].

## 4. Conclusions

Polycrystalline graphite is an inherently brittle material, which is known to exhibit ‘hardening’ behavior upon radiation damage. Most of the literature is dedicated to macro-scale mechanical property measurement as well as multi-scale defect characterization. There remains a knowledge gap at the submicron–nanoscale on the deformation mechanisms following exposure to radiation. Motivated by this research need, micropillar compression tests were performed on both pristine and ion-irradiated specimens. A 2.8 MeV Au^2+^ ion irradiation to a fluence of 4.378 × 10^14^ cm^−2^ (~peak 8.8 dpa) was used to produce cascade damage similar to that created by neutron radiation. The experiments were performed inside a TEM so that microstructural visualization could be performed in real time as the specimens were loaded and unloaded.

Several features of the loading and unloading were observed that are unusual in the graphite literature. First, the radiated specimen did not show any appreciable increase in stiffness or strength compared to the pristine counterparts. Second, the specimens showed remarkable plastic deformation. In fact, all irradiated specimens bulged but did not fracture. This is in stark contrast with the pristine specimens, all of which fractured catastrophically. Third, the plastically deformed specimen showed numerous kink bands that appeared to be moving along a slip plane (45° to the direction of loading), similar to dislocations piling up against a grain boundary in metallic materials. Fourth, the defect mobility is significantly high as evident from the live video recording. Finally, a part of the applied displacement recovered, suggesting the deformation was not completely elastic. It is reasonable to expect a strong size effect in the specimens, as commonly reported in the literature. However, our findings do not match the trend of ‘smaller is stronger’ or ‘smaller is tougher,’ which are commonly shown for unirradiated specimens. In this study, our pristine specimen behavior is consistent with the literature, and therefore it does not show any size effect. Comparing the irradiated specimen behavior with the pristine, the conventional point defect models are unable to explain the observed behavior. Rather, more recent models employing ripplocations and kink bands are more consistent with our in situ TEM observations.

## Data Availability

Data can be obtained by email request.

## Supplementary Materials

The following supporting information can be downloaded at: https://www.mdpi.com/article/10.3390/ma17040895/s1, Supplementary Information (pdf): video files of micropillar compression tests (zip).
